# Extremophiles and Extreme Environments: A Decade of Progress and Challenges

**DOI:** 10.3390/life14030382

**Published:** 2024-03-14

**Authors:** Pabulo Henrique Rampelotto

**Affiliations:** Bioinformatics and Biostatistics Core Facility, Institute of Basic Health Sciences, Federal University of Rio Grande do Sul, Porto Alegre 91501-970, Brazil; prampelotto@hcpa.edu.br

## 1. Introduction

It is with great enthusiasm that we embark on a retrospective journey to the landmark work “Extremophiles and Extreme Environments” (1 in Appendix A), published 10 years ago as part of its Special Issue (2 in Appendix A). As a testament to its enduring impact, that article stands as the most cited within the esteemed journal *Life* and holds a prestigious position in the literature, trailing only behind *Nature*’s 2001 “Life in Extreme Environments” (3 in Appendix A).

That seminal work, along with its accompanying Special Issue, not only marked a watershed moment but also paved the way for several Special Issues and numerous papers in MDPI journals, exploring the intriguing world of extremophiles. Since that pioneering work, this field has witnessed remarkable advances while also encountering new challenges. In the spirit of honoring past triumphs and navigating the uncharted territory that lies ahead, this editorial emerges as a dual-purpose narrative: a celebration of past achievements, and a strategic roadmap for future endeavors.

As we reflect on the past decade’s accomplishments and challenges, this editorial aims to inspire future research, collaboration, and innovation in the fascinating field of extremophiles and extreme environments. To illuminate the trajectory of progress, I first discuss ten significant developments that have occurred in this field during the past decade. At the same time, I also explore ten major challenges that require our collective attention and innovative solutions.

## 2. Ten Major Advances in Extremophile Research

Over the past decade, the field of extremophile research has witnessed significant advances, driven by technological innovations and interdisciplinary collaborations. From uncovering novel extremophile species to elucidating the molecular mechanisms of adaptation as well as new biomedical applications, these advances have expanded our understanding of life’s resilience, diversity, and biotechnology potential. In this section, I explore the ten major advances in extremophile research over the last decade, highlighting their impact and implications for this field.

### 2.1. New Methods for Culturing and Studying Extremophiles

Recent advances in extremophile research have led to the development of innovative methods for culturing and studying these organisms in laboratory settings. These methods include the use of microbial fuel cells, high-throughput screening, and advanced imaging techniques, which have facilitated more efficient and effective study of extremophile biology and genetics. By enabling extremophiles to be grown under less-extreme conditions, these new culturing techniques have expanded our ability to investigate these fascinating organisms, with significant implications for biotechnology, environmental science, and astrobiology.

### 2.2. The Omics Revolution

The Omics Revolution, driven by advancements in sequencing technologies and metagenomics, has revolutionized extremophile research. This transformative phase has led to the discovery and detailed characterization of novel extremophile species, expanding our understanding of the diversity of life on Earth. Furthermore, the study of extremophile genomes, transcriptomes, proteomes, and metabolomes has revealed key genes, proteins, and metabolites involved in stress tolerance, offering valuable insights into extremophile adaptation with potential applications in biotechnology and industrial processes, such as the production of biofuels. This revolution has significantly advanced our knowledge of extremophiles and their unique adaptations, opening up new avenues of research and applications in biotechnology.

### 2.3. Ecology and Diversity of Extremophiles

The ecology of extremophiles is a complex and crucial area of study with significant implications for ecosystem dynamics and ecological balance. Extremophiles play essential roles in biogeochemical cycles and ecosystem functioning in extreme environments, contributing to nutrient cycling and energy flow. Their ability to occupy niches inhospitable to other forms of life allows them to fulfill unique ecological roles, such as decomposer, primary producer, or predator. Extremophile communities exhibit complex interactions and relationships that influence ecosystem dynamics, including symbiotic relationships with other organisms. Additionally, extremophiles play vital roles in bioremediation, using their unique enzymes and metabolic pathways to clean up contaminated sites. Our continuous understanding of extremophile ecology has provided valuable insights into ecosystem dynamics and the mechanisms by which organisms interact with their environment.

### 2.4. Evolution of Extremophiles

Driven especially by the Omics Revolution (previously discussed), our understanding of the evolution of extremophiles has significantly expanded over the last decade. The peculiar evolution of such intriguing microbes involves the acquisition of unique genetic traits that enable them to thrive in extreme conditions, including specialized proteins and enzymes that function optimally under harsh environmental factors. Horizontal gene transfer (HGT) also plays a significant role in extremophile evolution by facilitating the acquisition of new genes or gene variants that provide adaptive advantages. Additionally, extremophile evolution is shaped by selective pressures in their habitats, driving them to develop survival strategies such as metabolic flexibility, dormancy, and symbiotic relationships. These insights have broader implications for evolutionary biology and genetics, enhancing our understanding of how organisms evolve and adapt to changing environments.

### 2.5. Environmental Exploration

The advances in exploration techniques have expanded our understanding of extremophiles’ diverse habitats, shedding light on life in extreme environments. These environments include the deep subsurface, high-altitude environments, hypersaline environments, hydrothermal vents, polar environments, as well as emerging categories such as anthropogenic extreme environments, technological extremes, acid mine drainage, cave systems, and subglacial environments. Studying extremophiles in these diverse habitats has provided valuable insights into the impact of human activities on microbial ecosystems and the potential for life beyond Earth, for example.

### 2.6. Astrobiology Insights

The study of astrobiology has expanded our exploration beyond Earth, with researchers investigating extreme conditions on celestial bodies such as Mars, Europa, and Enceladus to assess their potential for harboring life. Assessing habitability involves evaluating the presence of liquid water, potential energy sources, and the search for organic molecules. The study of extremophiles on Earth has provided valuable insights into the adaptability of organisms to extreme environments, serving as analogs for potential life forms that may have evolved unique strategies to survive in extreme conditions found elsewhere in the universe.

### 2.7. Biotechnological Applications

The ability of extremophiles to thrive in harsh conditions makes them promising candidates for sustainable and environmentally friendly biotechnological applications. These biotechnological applications have significantly advanced over the last decade, leading to several implications for biotechnology. Firstly, advancements in bioprospecting and genomic analysis have facilitated the discovery and characterization of novel extremophiles and their enzymes, expanding the pool of biocatalysts available for industrial processes. This has led to the development of more efficient and sustainable biotechnological processes, with implications for industries such as bioremediation, biofuels, and pharmaceuticals. Moreover, the engineering of extremozymes for specific industrial applications has been enhanced through protein engineering and directed evolution techniques, leading to the creation of enzymes with tailored properties for various biotechnological processes. These developments have improved the efficiency and cost-effectiveness of industrial processes, with implications for the production of biofuels, pharmaceuticals, and other bioproducts.

### 2.8. Medical Applications

Extremophiles offer significant potential for medical applications, as their unique adaptations make them a valuable source of bioactive compounds and enzymes with antimicrobial, anticancer, and immunomodulatory properties. These medical applications of extremophiles have significantly advanced over the last decade, leading to several implications for medicine. Advances in bioprospecting and genomic analysis have enabled the discovery and characterization of bioactive compounds and enzymes from extremophiles, expanding the potential for developing novel drugs and therapies. This has led to the exploration of extremophiles as a source of new antibiotics, anticancer agents, and immunomodulatory compounds, offering potential treatment options for infectious diseases, cancer, autoimmune diseases, allergies, and other immune-related disorders.

In addition, the study of extremophiles has contributed to the understanding of unique biochemical pathways and molecular mechanisms that underlie their adaptations to extreme environments. This knowledge has provided insights into the development of innovative pharmaceuticals and therapeutic strategies, with implications for drug discovery, personalized medicine, and the treatment of complex diseases. Furthermore, the use of extremophiles in medical applications has the potential to address challenges such as antibiotic resistance and the need for novel therapeutic agents. By exploring extremophiles as a source of bioactive compounds, researchers have the opportunity to develop alternative treatments for conditions that are difficult to manage with existing therapies.

### 2.9. Synthetic Biology

Synthetic biology has become a powerful tool for extremophile research, allowing the design and construction of biological systems with novel functions or capabilities. By engineering extremophiles, researchers have developed biotechnological applications for purposes such as bioremediation or bioproduction. Advances in synthetic biology have also facilitated the creation of synthetic extremophiles with enhanced stress tolerance or novel functionalities, offering new possibilities for biotechnological applications, such as the production of new materials or the development of resilient crops.

### 2.10. Collaborative Initiatives

Collaborative research initiatives have significantly advanced extremophile research by fostering interdisciplinary studies and bringing together researchers from various scientific disciplines. This collaborative approach allows scientists to combine their expertise and perspectives, leading to a more comprehensive understanding of extremophiles and their unique adaptations. Extremophiles have captivated the interest of biologists, chemists, geologists, astrobiologists, and other scientists, highlighting the need for a collective approach to unravel the intricacies of extremophiles. Collaborative initiatives in extremophile research facilitate the integration of diverse perspectives and methodologies, leading to a more holistic understanding, as well as enabling the pooling of resources, expertise, and data for more efficient and impactful research outcomes.

## 3. Ten Ongoing Challenges in Extremophile Research

As our investigation into extremophiles progresses, we also encounter a myriad ongoing challenges that shape the trajectory of this field. In this section, I explore the ten main challenges in extremophile research that I consider to be the most relevant over the last ten years. Through my perspective, I hope to shed light on the complexities and intricacies that come with uncovering the secrets of these extraordinary organisms.

### 3.1. Sampling and Isolation

One of the primary ongoing challenges in extremophile research involves sampling and isolating these organisms from their extreme habitats, such as deep-sea hydrothermal vents, polar ice caps, and acidic hot springs. Accessing these environments can be a logistical and technical hurdle for researchers, requiring specialized equipment and expertise. Innovative techniques are required to collect extremophile samples while minimizing contamination and preserving the integrity of the organisms. Once collected, isolating extremophile organisms from their complex environmental matrices can be challenging, as they often exist in low abundance and may be difficult to culture in the lab. We are increasingly using molecular techniques to identify and isolate extremophiles, allowing us to identify previously unknown species and gain insight into their genetic makeup and unique adaptations.

### 3.2. Cultivation and Growth Conditions

Cultivating and studying extremophiles in laboratory settings presents a significant challenge due to their specific growth requirements, such as extreme temperatures, pH levels, or salinity. Recreating these conditions in the lab is crucial for successfully growing and studying these organisms. We must use specialized media and growth conditions that mimic the organisms’ natural habitats, and carefully optimize these growth conditions to ensure the survival and growth of the extremophiles. Despite the challenges, the potential applications of the bioactive compounds and enzymes produced by extremophiles make the cultivation process rewarding.

### 3.3. Taxonomic Classification

The taxonomic classification of extremophiles presents significant challenges due to their unique genetic makeup and adaptations. Traditional classification methods may not adequately capture the diversity and evolutionary relationships among extremophiles. Developing robust taxonomic frameworks that encompass the genetic, physiological, and ecological characteristics of extremophiles is crucial for understanding their evolutionary history and ecological roles. Challenges include the lack of reference genomes and genetic data, reconciling genetic and physiological characteristics with traditional taxonomic categories, understanding ecological roles, and considering the ethical and philosophical implications of defining life. Overcoming these challenges will require innovative research approaches, interdisciplinary collaborations, and technological advancements.

### 3.4. Mechanisms of Adaptation

Understanding the mechanisms by which extremophiles adapt to extreme environments is a fundamental challenge in extremophile research. Extremophiles have evolved specialized adaptations that allow them to thrive in conditions inhospitable to most other organisms, such as thermophilic enzymes, osmolyte production, and DNA repair mechanisms. Studying these mechanisms presents challenges related to accessing extremophile organisms and their habitats, studying extremophile adaptations in the laboratory, understanding the genetic and biochemical basis of extremophile adaptations, and integrating techniques from various disciplines to gain a comprehensive understanding of extremophiles’ coping mechanisms with extreme environments. Overcoming these challenges is crucial for gaining insights into the limits of life and harnessing the potential of extremophiles for various applications.

### 3.5. Genetic Plasticity

Understanding the genetic plasticity of extremophiles, which enables them to rapidly adapt to changing environmental conditions, presents significant challenges. These challenges include studying extremophile genomes, deciphering unique genetic elements specific to extreme habitats, understanding regulatory mechanisms for gene expression in response to environmental stressors, and decoding complex gene clusters and regulatory networks. Additionally, harnessing extremophile adaptations for practical purposes requires a deep understanding of the underlying genetic mechanisms and the ability to manipulate and engineer extremophile genomes.

### 3.6. Exploring Ecological Interactions

Studying the ecological interactions of extremophiles within their extreme habitats is a challenging task due to the complex nature of these environments. Extremophile communities have intricated symbiotic relationships and nutrient-cycling processes that contribute to the functioning of their ecosystems. However, accessing and studying these extreme environments, as well as untangling and understanding the complexity of the interactions, pose significant challenges. Despite these obstacles, investigating ecological interactions among extremophiles is crucial for understanding the functioning of extreme ecosystems and developing strategies for conserving and protecting these unique environments.

### 3.7. Metabolic Engineering Complexity

Understanding and modifying the intricate metabolic pathways of extremophiles for specific biotechnological applications presents an ongoing challenge due to the complex nature of extremophile metabolic networks and specialized adaptations. Extremophiles have evolved unique biochemical pathways and enzyme systems to thrive in extreme environments, and modifying these pathways to enhance the production of desired compounds or confer specific traits is a significant challenge. This complexity requires a deep understanding of extremophile physiology, genetics, and metabolic regulation, as well as advanced tools for genetic manipulation and pathway optimization. Addressing the metabolic engineering complexity of extremophiles requires innovative approaches, advanced computational tools, and interdisciplinary collaboration to unravel and manipulate their intricate metabolic networks for various biotechnological applications.

### 3.8. Standardization of Medical Applications

The standardization of medical applications for extremophiles is an ongoing challenge due to the diverse nature of extremophile-derived compounds and their potential medical uses. Extremophiles produce a wide variety of bioactive molecules, enzymes, and metabolites that could have therapeutic applications, ranging from antimicrobial agents to enzymes for diagnostic and therapeutic purposes. Standardizing these applications involves ensuring consistent quality, safety, and efficacy of the products derived from extremophiles. This includes developing standardized protocols for extraction, purification, and characterization of extremophile-derived compounds, as well as establishing regulatory frameworks to evaluate their safety and efficacy for medical use. Standardization is crucial for ensuring reproducibility and comparability of results across different studies and for the eventual translation of extremophile-derived compounds into clinical applications. As such, this ongoing challenge requires interdisciplinary collaboration and rigorous scientific validation to harness the potential of extremophiles for medical purposes.

### 3.9. Extreme Habitat Replication

Accurately replicating extreme habitats is crucial for studying extremophiles, understanding their adaptations, and harnessing their unique capabilities for biotechnological applications. However, this presents significant technical challenges due to the difficulty in reproducing the extreme environmental conditions in which these organisms thrive. For example, mimicking high temperatures, acidic conditions, high-pressure environments, or extreme radiation conditions can be technically demanding and costly. Addressing this ongoing challenge requires the development of innovative technologies and methodologies to accurately mimic and study these extreme environments in laboratory settings.

### 3.10. Climate Change Impacts

Understanding how extremophiles respond to environmental perturbations is crucial for predicting their resilience and assessing the potential implications for ecosystem dynamics. However, assessing the impacts of climate change on extreme environments poses significant challenges. These challenges include limited baseline data for comparison, deciphering complex interactions between climate change and extreme environments, conducting long-term observations and experiments, understanding interconnected ecosystems, and predicting the future trajectory of climate change and its impacts on extreme environments. Overcoming these challenges is crucial for gaining insights into extremophile responses to environmental perturbations and assessing their resilience and potential implications for ecosystem dynamics in the face of climate change.

## 4. Final Remarks

Extremophiles represent a unique and fascinating area of research with significant implications for medicine, biotechnology, astrobiology, and environmental science. The study of extremophiles has advanced significantly over the past few decades, with numerous technological and methodological advancements enabling us to explore these extreme environments and the intriguing microbes within them. However, the field of extremophile research also faces significant challenges, including accessing extremophile habitats, replicating extreme conditions in laboratory settings, and interpreting findings in the context of astrobiology or climate change. Overcoming these challenges will require interdisciplinary collaborations, innovative research approaches, and technological advancements. Nevertheless, the potential insights gained from studying extremophiles make this area of research essential for advancing our understanding of life on Earth and beyond.

## Appendix A

1. Rampelotto, P.H. Extremophiles and Extreme Environments. *Life* **2013**, *3*, 482–485. https://doi.org/10.3390/life3030482.
2. Available online: https://www.mdpi.com/journal/life/special_issues/life-extremophiles (accessed on 30 January 2024).
3. Rothschild, L.J.; Mancinelli, R.L. Life in Extreme Environments. *Nature* **2001**, *409*, 1092–1101. https://doi.org/10.1038/35059215.

